# Characteristics and Welfare of Long-Term Shelter Dogs

**DOI:** 10.3390/ani11010194

**Published:** 2021-01-15

**Authors:** Christina Raudies, Susanne Waiblinger, Christine Arhant

**Affiliations:** Institute of Animal Welfare Science, Department for Farm Animals and Veterinary Public Health, University of Veterinary Medicine, Vetmeduni Vienna, Veterinärplatz 1, 1210 Vienna, Austria; susanne.waiblinger@vetmeduni.ac.at

**Keywords:** dog behaviour, animal shelter, animal welfare, stress, adoption, relinquishment

## Abstract

**Simple Summary:**

In no-kill shelters, overpopulation is an often-faced problem. Some individuals have better adoption chances than others, and over time, long-term dog populations develop. Our aim was to identify certain characteristics that long-term shelter dogs share and to investigate if long-term shelter dogs experience an impairment of welfare due to the restricted environment. In our study, long-term shelter dogs were more often of older age, male, of large size, neutered, and of a “dangerous breed”. They were also described more often as having behavioural problems regarding aggression and high arousal. The physical wellbeing of long-term shelter dogs was not impaired. However, they did show some stress-related behaviours, suggesting that they might be more affected by acute stressors and have more difficulties relaxing in the shelter environment. In sum, certain morphological and behavioural characteristics of dogs can be used to identify individuals at higher risk for a long-term stay. Thus, these dogs require special attention and effort to enhance their adoption chances. The results of this study serve as a scientific basis for developing such dog-specific strategies.

**Abstract:**

To identify characteristics that distinguish long-term (LT: stay > 1 year) from short-term shelter dogs (ST: ≤5 months) and to investigate if a long-term stay impairs welfare, we compared ST and LT dogs in Austrian no-kill shelters. Analyses including characteristics such as breed, sex, or age (shelter records), problem behaviour, and personality (questionnaires completed by staff) showed that LT dogs were significantly more often a “dangerous breed”, male, and older when admitted to the shelter. They were rated higher on “aggression” and “high arousal” and lower on the personality dimension “amicability”. A welfare assessment protocol including reaction toward humans (Shelter Quality Protocol), and in-kennel observations were used to assess the effect of the long-term stay. LT dogs tended to show more signs of aggression toward an unfamiliar human, but welfare assessment revealed no difference. During resting periods, LT dogs spent more time resting head up and had more bouts resting head down. Prior to feeding, they stood, vocalised, and yawned more. LT dogs are characterised by specific features such as being aroused easily and having difficulties to relax. Whether this is a result of the long-term stay or personality-associated, consequently causing lower adoption rates, remains to be determined.

## 1. Introduction

The general purpose of an animal shelter is the temporary housing and re-homing of unwanted, abandoned, and stray animals [1]. If a no-kill policy is practiced, as is legally required in Austria, some dogs may have a prolonged stay in the shelter, and over time, a population of long-term shelter dogs develops. Several studies revealed physical or other characteristics of dogs that were associated with (reduced) adoption rates and thus might influence the length of stay of dogs in shelters. The appearance of the animal, its personality, and the behaviour towards the potential adopter were the most important factors influencing adoption in a study in the USA [2]. Studies about preferred characteristics have not been consistent, which might reflect regional differences in preferences, but the following characteristics were often linked to a prolonged stay in shelters: found/stray dogs [3,4], older age [5], larger size [5,6,7], being sexually intact [8,9], being male [8,9,10,11,12], having dark or brindled coat colour [6,12,13], having short-haired coat [14], being of mixed breed [6] or being a member of the so-called “fighting breeds” [12]. In addition to those characteristics, an adopter’s choice may further be built on information on (problem) behaviour and a dog’s personality. An adopter survey revealed that the adoption chances increase if the dog shows affiliative behaviour when first making contact [2]. Although high arousal and problems with controlling a dog’s behaviour (e.g., pulling on the lead, chasing things, stealing food) are the most frequently reported undesirable behaviours by dog owners, aggression was named as the most problematic behaviour for dog owners [15]. In general, dog owners prefer personality traits such as playfulness, friendliness, sociality, obedience, affection, compliance, high energy levels, and non-aggressiveness [2,16]. In Austria in 2009, almost one-third of the dogs housed in shelters were considered difficult to rehome by the shelter manager [17]. Despite this high proportion of potentially long-term housed dogs, to date, there has been no information about dog characteristics influencing the length of stay of Austrian shelter dogs. Therefore, in the first part of the study, we aimed at identifying the characteristics that differentiate long-term from short-term shelter dogs in Austrian shelters. We hypothesise that the group of long-term shelter dogs share certain morphological and behavioural characteristics that distinguish them from those dogs that could be rehomed within a short period of time. We also expect that reports on long-term shelter dog’s behaviour show more different or more severe forms of problem behaviours and that they will be ranked lower in the favoured personality traits compared to short-term housed dogs.

Confinement in a shelter environment often prevents a dog from engaging in species-specific behaviour such as the exploration of new environments and contact to conspecifics [18]. In Austrian shelters, 66% of the dogs are kept singly [17]. Furthermore, shelter dogs are confronted with a new and often unpredictable environment, and they experience a loss of control [19]. In the long run, this may cause chronic stress and lead to decreased welfare in sheltered dogs [19,20]. Socially and spatially restricted dogs showed increased aggression, excitement, and uncertainty when challenged with an acoustic stressor [21]. Behaviours such as paw lifting, vocalisations, repetitive behaviours [20], circling, self-licking, panting [22], and holding the head up during resting [23] were reported as signs of chronic stress and may therefore be used as indicators for impaired welfare. Previous studies looking into the welfare of shelter dogs most often did not compare the welfare status of long-term and short-term shelter dogs (e.g., [22]), and if this distinction was made, the long-term group comprised individuals with a length of stay of up to several weeks [24]. However, in Austrian shelters the average length of stay of a shelter dog is 5 months [17] rather than several weeks, and some dogs are housed for more than a year or even for many years. Based on that, we considered dogs housed up to 5 months as short-term shelter dogs and dogs that were housed in the shelter for at least one year as long-term shelter dogs. Therefore, the second aim of our study was to explore possible effects of the long-term confinement on the dogs’ behaviour and welfare. We hypothesise an increase in chronic stress levels in long-term shelter dogs indicated by more common occurrence of welfare problems as indicated by a welfare assessment tool including approach behaviour towards humans [25,26,27] and a greater prevalence of stress-related in-kennel behaviours and hence an overall decrease of welfare compared to short-term shelter dogs.

## 2. Materials and Methods

### 2.1. Ethical Statement

This study (ETK-09/04/2018) was discussed and approved by the institutional ethics and animal welfare committee in accordance with ‘Good Scientific Practice’ guidelines (GSP) and national legislation.

### 2.2. General Study Design and Shelters

This study consists of two parts. Part I investigated the general, morphological, and ethological characteristics relative to the length of stay in the shelter. In Part II, the dogs’ welfare and behaviour in relation to the length of stay was assessed. The study was performed in June 2018 until August 2018.

For both parts, the dogs were classified as “long-term” dogs (LT) or “short-term” dogs (ST) according to their length of stay in the shelter. The ST group was characterised by a length of stay up to five months. The maximum stay of five months represents the median length of stay in Austrian shelters [17]. Individuals of the LT group had a minimum length of stay of one year. This categorisation criterion was chosen as Austrian shelters are allowed to go below the legal requirements for the dogs’ housing conditions up to one year as long as dogs do not show behavioural alterations and their adaptation capacity is not overextended [28]. Theoretically, this concerns all legally regulated aspects such as resources and daily care. However, in practice, mainly space allowance may differ if a shelter has kennels smaller than 15 m^2^, which is the required minimal space for one dog in Austria. In none of the shelters included in the study, total space allowance including indoor and outdoor sections of kennels was smaller than 15 m^2^. After one year of stay in the shelter, housing is no more considered to be temporary; therefore, the usual legal requirements for keeping a dog must be fulfilled.

For feasibility reasons, we reached out to shelters in the surrounding of Vienna (up to around 100 km distance). In total, nine shelters were contacted, five of them declined our request mainly due to human resource constraints and the concern of the implication of our study protocol into the daily shelter routine. Therefore, four shelters participated. Two of them were located in Lower Austria in medium-sized cities (25,000/55,000 inhabitants), and the other two were located in Vienna (1.9 million inhabitants). Data from shelter records were collected in all four shelters, whereas questionnaire and behavioural data could only be collected in three shelters. At the start of data collection in the respective shelters, the four shelters were housing 30 dogs (11 LT, 10 ST), 37 dogs (15 LT, 13 ST), 162 dogs (17 LT, 108 ST), and 138 dogs (61 LT, 56 ST). On the first day of a shelter visit, the data from the shelter records for part I regarding general and morphological characteristics were acquired. For assessment of personality as well as behavioural problems (part I) and dog in-kennel behaviour and welfare (part II), shelters had to be visited several times depending on the number of dog pairs assessed at that shelter. The personality and behavioural problem questionnaires were handed to the shelter staff on the day of assessment of a dog pair. One pair per day was assessed. The evaluation of behavioural problems and personality of the dogs in part I as well as the in-kennel behaviour and welfare assessment in part II were based on a matched pairs design [29]. Thereby, we aimed at controlling for differences in basal stress levels and management between shelters and any differences in behaviour that could arise from the age or the breed of the dog to only detect differences between dogs due to the length of stay of the dogs in the shelter. All LT dogs, which were offered for adoption, were used for this study. Some shelters excluded dogs that would have become excessively excited and stressed due to the study’s procedure. The investigator (CR) matched the LT dogs as closely as possible for housing condition (space allowance), sex, breed/type of dog, and age with an ST dog. Only single housed dogs were included in this study. For part I (personality and behavioural problems), the LT dogs were paired with ST dogs that had been already rehomed. For part II, the same LT dogs were paired with ST dogs still staying in the shelter to allow comparison of welfare assessment and in-kennel observations. All ST dogs used in the matched pairs design had a minimum stay of two weeks to avoid assessing potential acute stress responses in the dogs that could have influenced the assessment of problem behaviour, personality, or behavioural observations including approach towards humans [14,27]. The dog pairs with the main pairing criteria are shown in the supplementary material (Supplementary Excel S1).

### 2.3. Part I: General and Morphological Characteristics, Problem Behaviour, and Personality

#### 2.3.1. General and Morphological Characteristics

Information about breed, size, coat characteristics, sex, neuter status, age, and mode of relinquishment was collected using the shelter records for all dogs that were currently housed in the shelter or that had entered or left the shelter within the last 365 days starting from the day of the data collection at the respective shelter, except for cases in which dogs were returned to the owner. In total, information on 1560 dogs was collected of which 1299 were ST dogs, 141 were LT dogs, and 120 were intermediate dogs (IM, length of stay between five months and one year). The gap between the ST and the LT population was intended to create two clearly distinct groups. Dogs with intermediate length of stay (IM dogs) were excluded from the analysis as well as ST dogs that currently stayed at the shelter. Data of all LT dogs and only data of 1111 ST dogs that were already rehomed were used for analyses.

The age of currently sheltered dogs was calculated using the duration between day of birth (or estimated day of birth) and day of the data collection. The age of already rehomed dogs at the time of data collection was calculated from the day of birth up to the day of adoption. The age when being admitted to the shelter was calculated from the day of birth up to the day of shelter admission. If no information about the day of birth or the day of shelter admission was available, these individuals were excluded from the statistical analysis regarding age.

The dogs’ size was categorised according to seven groups: XXS (<25 cm height at the shoulder), XS (26–31 cm), S (32–36 cm), M (37–41 cm), L (42–46 cm), XL (47–51 cm), and XXL (>52 cm). If there was no information about the dog’s size in the shelter records, the size was estimated from pictures or from the average standard size of the dog’s breed. If the dog was a mix, the average standard size of the first listed breed for this dog was applied.

The breeds were categorized into “purebred”, “mix” and “dangerous breed”. The purebred group included all breeds except for “dangerous breeds”, whilst the mix group included all mixed breed dogs except mixes of the “dangerous breeds”. The so-called “dangerous breeds” were included as a discrete group because a breed-specific legislation exists in the areas where the shelters are located. It included all breeds and their mixes that were listed in the breed-specific legislation in the county where the data were collected. In Vienna, the following breeds were listed: Bull terrier, Staffordshire Bull Terrier, American Staffordshire Bull Terrier, Mastino Napolitano, Mastin Espanol, Fila Brasiliero, Mastiff, Bull Mastiff, Tosa Inu, Pit Bull Terrier, Rottweiler, Dogo Argentino, and Bandog [30]. In Lower Austria, the following breeds were listed: Bull Terrier, Staffordshire Bull Terrier, American Staffordshire Bull Terrier, Tosa Inu, Pit Bull Terrier, Rottweiler, Dogo Argentino, and Bandog [31].

Information about the coat included colour and hair length. The coat colour was categorised as light (white, gold, light brown, or any mix of these), dark (black, dark brown, grey, or any mix of these), multicolour (two or more colours out of the light and dark group) or brindled. The length of the coat was divided into five different categories as followed: short (e.g., Staffordshire bull terrier), medium (e.g., German shepherd), long (e.g., Golden Retriever), curly (e.g., Poodle), and wirehaired (e.g., Schnauzer). If there was no information about the coat length within the shelter records, the common coat length of the dog’s breed was applied. Mixed breed individuals’ coat length was gathered from the first breed documented for this dog. If no information about the coat colour or length or the dog’s breed was available, these dogs were not included in the statistical analysis regarding the coat.

The sex of each dog was noted as well as the neuter status. If no information about the dog’s sex was available within the shelter records, the sex was deduced from the dog’s name, if possible.

Four distinct modes of relinquishment were differentiated within this study: found (stray dogs), owner-surrendered (including owner’s demise, their hospitalisation, or jail time), confiscation by authorities, and birth in the shelter.

#### 2.3.2. Problem Behaviour and Personality

This part of the study was conducted with 24 dog pairs of LT dogs matched with already rehomed ST dogs. The chosen rehomed ST dogs were individuals the shelter staff could remember vividly. Using rehomed ST dogs ensured that their maximum length of stay did not exceed the five months. In order to assess behavioural problems, the shelter staff member that knew the dog best from handling on a daily basis was asked to rate each dog’s behaviour on a four-point scale (1 = never, 2 = occasionally, 3 = sometimes, 4 = frequently). In order to avoid language barriers, the questionnaire was translated into German. The shelter staff person was also asked to judge if the behaviour in question might jeopardise the dog’s rehoming. The questionnaires were answered by one staff person (who was the main caretaker) per shelter for all pairs to avoid bias from different raters.

Personality assessment was conducted with the same 24 pairs used for the assessment of behavioural problems. The revised version of the Monash Canine Personality Questionnaire (MCPQ-R) from Ley et al. (2008) [32] was used. In addition, this questionnaire was translated into German to prevent any language barriers. The questionnaire was answered by the same staff member who completed the problem behaviour questionnaire. Both questionnaires were given to staff members on the day of the dog pair’s assessment to complete them when their time allowed.

### 2.4. Part II: Welfare Assessment

Welfare of the dogs was assessed by use of the Shelter Quality Protocol (SQP) [25], which was used to measure reactions towards an unfamiliar human [27] and behavioural observations during a resting and a pre-feeding period. This part was conducted with 20 matched pairs of LT and ST dogs currently housed in the shelter (Supplementary Table S1). One matched pair was assessed per day. The unfamiliar-human-test and the Shelter Quality Protocol were first completed for one dog before testing the matched one. It was randomised whether the LT or the ST dog of a pair was tested first. The first recording for the behavioural observations took place before feeding, the second took place during a resting period. Video recordings were done simultaneously for each dog pair. Assessments were conducted in the following order: unfamiliar person tests, SQP, and recordings of behaviour.

For the assessment of the reaction towards an unfamiliar human, a combination of the unfamiliar human test in [27] and the test for reaction towards humans from the SQP [25] was used. The investigator (CR) stood motionless 2 m away from the kennel for 30 s. If a distance of 2 m was not possible, the investigator started at the largest distance possible to the kennel. Then, the investigator approached the dog’s kennel door at a 90° angle with slow and smooth movements and the arms hanging down with a speed of 1 step per second, the gaze focussing on the floor, and stopped approximately 30 cm in front of the door. The investigator raised her right arm until the back of the hand was approximately 5 cm from the kennel door and left her hand there for 5 s. It was noted if a contact was “possible” or not “possible” according to [27]. “Contact possible” was defined when the dog approached and explored the experimenter; dogs showing approach and or exploration behaviour toward the person but barked or growled intermittently were also rated as “contact possible” [27]. Then, the investigator lowered the arm, kneeled down and talked to the dog in a friendly voice for another 30 s. The dog’s approach behaviour was observed and classified as “no signs”, “fear” or “offensive/defensive aggression” according to the SQP [25]. During the whole procedure, the investigator avoided staring at the dog, and no physical contact was established.

After the reaction to the unfamiliar person was assessed, the animal- and resource-based parameters of the SQP were recorded, except for the assessment of coughing and abnormal behaviour [25].

Regarding behavioural observations, each pair was video recorded twice for 20 min on the same day. To ensure the same recording conditions, a pair was recorded simultaneously with one camera per dog. One recording was taken before feeding (pre-feeding period) and one recording was taken during a resting period. After installation and starting of video recordings, the experimenter was not present during the recordings. In order to reduce potential stress for tested individuals during camera setup, dogs were transferred if possible into the adjacent outdoor enclosure throughout the installation of the portable cameras and the attached computer system (SANYO Electric Co. Ltd., Moriguchi Japan 1/3”, VCC-HD2300P Full HD 1920 × 1080 (POE) Power over ethernet 2.8–8 mm Optic; software GeoVision Surveillance System V8.5.6). The 20 min videos were coded with the Behavioural Observation Research Interactive Software (BORIS) v.6.3.6 [33]. The ethogram listed in Table 1 gives the definitions of observed behaviours. Continuous behaviour coding was used. Intra-observer reliability was analysed using the first 5 min of a random selection of 12 videos, which were coded twice with a time lag of two weeks. In order to assess the inter-observer reliability, the same 12 videos were coded by an independent person who was blinded to the group affiliation of the dogs. The investigator (CR) was not blinded for the dogs’ group affiliation, as she matched the pairs and was therefore aware of the dogs’ length of stay. Data of the intra-observer reliability and the inter-observer reliability tests were analysed using a Spearman correlation and a subsequent Wilcoxon signed-rank test. The correlation coefficient rs was categorised as following: <0.2 “slight”, 0.2–0.39 “low”, 0.4–0.69 “moderate”, 0.7–0.89 “high”, and 0.9–1.0 “very high” [34]. The correlation of the intra-observer reliability for durations was ranging from r_s_ = 0.993 to r_s_ = 1 (mean r_s_ = 0.999) and for frequencies from r_s_ = 0.998 to r_s_ = 1 (mean r_s_ = 0.998). The Wilcoxon signed-rank test revealed a directed difference (*p* < 0.05) for the duration of resting with head down, with consistently longer duration the second time a video was analysed. However, the mean difference in duration was only 0.3 s, and thus, we decided to include it in the analyses. Except for the behaviour licking (duration r_s_ = −0.134, frequency r_s_ = −0.172), the inter-observer reliability was high to satisfactory (r_s_ = 0.844 to r_s_ = 1. The according results from the Wilcoxon signed-rank test for the durations and frequencies were ranging from *p* = 0.041 to *p* = 1 (mean durations = 0.785, mean frequencies = 0.570). A directed bias was revealed by the Wilcoxon signed-rank test for the behaviour standing (*p* = 0.041). The duration was rated consistently higher by one observer with at mean difference of 2 s. We did not exclude these behaviours (licking, standing), as the final data for analyses were coded by only one observer, and her intra-observer reliability was good. Nevertheless, the ethogram for these behaviours was refined to ensure reliable coding. The behaviours circling, hiding, chewing, and paw lifting were rare and did not occur in the video segments chosen for the inter- and intra-observer reliability test; therefore, reliability could not be calculated.

### 2.5. Data Analysis

All data were analysed using the software IBM SPSS Statistics for Windows (Version 23.0. IBM Corp: Armonk, NY, USA. IBM Corp. Released 2015), and the graphs were produced with the software R (Version 3.5.2, https://www.r-project.org/ [35]. As data were not normally distributed, non-parametric statistics were used.

In order to test for differences between the LT and the ST dogs in part I with respect to general and morphological characteristics on a metric scale, i.e., age and age when admitted, the Mann–Whitney U test was applied. Differences between the groups (LT/ST) regarding categorical general and morphological characteristics were examined by cross-tabulations and the Chi^2^-test; in case of a significant Chi^2^-test (*p* < 0.05), the observed frequency in cells with a standardised residual (SR) greater than 2 or smaller than −2 was considered to significantly deviate from the expected frequency [36].

For the behavioural problems and personality questionnaire as well as the in-kennel behavioural observations, we used a matched pairs design [29].

The items of the problem behaviour questionnaire were thematically grouped into the categories “aggression” (aggression towards dogs, aggression towards familiar persons, aggression towards unfamiliar persons, guarding food bowl, redirected aggression), “fear” (hiding from familiar persons, hiding from unfamiliar persons, fear of noise, fear of dogs), “inappropriate in-kennel behaviour” (destructive behaviour, barking, repetitive behaviour), “high arousal” (jumping, excitement about familiar persons, excitement about unfamiliar persons, excitement about food, excitement about walk), and “miscellaneous” (pulling on the leash, chasing bicycles, jogger etc., mouthing hands or clothes, sexual behaviour towards humans). For each dog, a mean score for each category was calculated by adding all the individual item scores per category and dividing it by the number of items. Differences of these scores within the matched pairs were examined using a Wilcoxon signed-rank test. In addition, one open question asked for other unwanted behaviours displayed by the dog. Results of the open question were analysed descriptively. In order to analyse the shelter staff members’ judgement, if the problem behaviours assessed in the questionnaire could jeopardise adoption, McNemar tests were conducted. The items of the personality questionnaire were grouped into the five dimensions “amicability”, “training factor”, “extraversion”, “motivation”, and “neuroticism” as proposed by Ley et al. (2008) [32], and the mean score per category was calculated. In order to test for differences within the matched pairs, a Wilcoxon signed-rank test was used.

In order to test for differences within pairs in the reaction towards an unfamiliar human (contact/no contact; fear/no fear; aggression/no aggression), McNemar tests were conducted.

The in-kennel behavioural observation data were corrected for the time the dogs were not visible by dividing the observed duration or frequency, respectively, by the time the dog was visible (in seconds) and multiplying by 600, thus representing duration or frequency per 10 min. Differences in corrected duration and frequency were once examined between the feeding and resting period within groups (LT and ST) and once examined within pairs (LT vs. ST) for both observation periods (resting, pre-feeding) by performing Wilcoxon signed-rank tests.

Results were considered to be statistically significant at *p* ≤ 0.05 and to be a trend when 0.05 < *p* ≤ 0.1. The significance and trend level were corrected with the Bonferroni correction. To this purpose, the significance level of 0.05 was divided by the number of tests that referred to a specific hypothesis (general and morphological characteristics: 9 tests; problem behaviour: 5 tests; personality: 5 tests; reaction towards unfamiliar human: 3 tests) resulting in the corrected significance level. The p-values and resulting significance levels are described in the respective results section.

For in-kennel behavioural observations, an exploratory approach was adopted. Therefore, results were not corrected for multiple testing.

## 3. Results

### 3.1. Part I: General and Morphological Characteristics, Problem Behaviour, and Personality

The total population in the investigated shelters included 1560 dogs, of which 83% (1299) were ST and 9% (141) were LT dogs. Most prominent in the total population were male dogs (45%, 19% unknown), mixed breeds (68%), dark or multicoloured coat (40% and 34% respectively, 26% unknown), short-haired dogs (30%, 47% unknown), and dogs larger in size (17% L, 12% XL, 40% unknown).

In comparison with the ST rehomed group (N = 1111), the LT population (N = 141) consisted of more dogs belonging to the “dangerous breeds” and their mixes and less dogs belonging to mixed breed dogs of other breeds (Table 2). The LT group comprised more males than females. Furthermore, in the LT dogs, more individuals were neutered (Table 2). The between group comparison for the morphological characteristics also showed that the LT group consisted of substantially less XXS and XS dogs and more M, L, and XXL sized dogs (Table 2). However, size was not independent from the breed group. The “dangerous breed group” had no dogs smaller than size L.

The LT dog population was on average older (LT: 7.3 ± 3.5 yr. vs. ST: 3.1 ± 3.4, n = 558, Z = −10.571, *p* ≤ 0.001) and also older when being admitted to the shelter (LT: 3.67 ± 2.86 yr. vs. ST: 3.00 ± 3.34 yr., n = 558, Z = −4.125, *p* ≤ 0.001). When categorised according to age, the ST population consisted of 37% of dogs younger than 1 year (percentage calculated based on cases with known age), 54% between 1 and 9 years, and 9% older than 9 years. As the line for a long-term shelter stay is above one year, no dog of the LT group was younger than 1 year. In the LT group, 67% of the dogs were between 1 and 9 years old and 33% were older than 9 years (see frequencies and percentages calculated based on sample including cases with unknown age in Table 3).

Coat colour and length as well as mode of relinquishment were not different between groups (Table 2).

**Figure 1:**
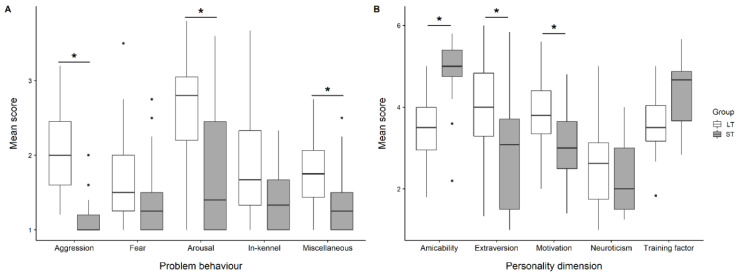
Comparison of LT and ST dogs’ problem behaviour and personality. (**A**)—Problem behaviour: Scores were 1 (never), 2 (occasionally), 3 (sometimes), and 4 (frequently). For each dog, a mean score was calculated by summing up the scores per category and dividing them by the number of behaviours; (**B**)—Personality assessment: Scores ranged from 1 (really does not describe the dog) to 6 (really describes the dog). The white boxes represent the long-term dogs, the grey boxes represent the short-term dogs. Results considered as statistically significant after correction for multiple testing are indicated with *.

With respect to problem behaviour, LT dogs had higher scores in the category “aggression” (mean ± SD: 2.1 ± 0.6 vs. 1.2 ± 0.4, *p* < 0.001, n = 24, (Figure 1A)**,** “high arousal” (2.6 ± 0.7 vs. 1.8 ± 0.8, *p* = 0.001, n = 24), and “miscellaneous” (1.8 ± 0.5 vs. 1.4 ± 0.5, *p* = 0.005, n = 24) than rehomed ST dogs. There was a trend with the LT dogs having higher scores in the category “inappropriate in-kennel behaviour” (1.8 ± 0.7 vs. 1.5 ± 0.4, *p* = 0.016, n = 24). No difference was found between groups in the category “fear” (1.6 ± 0.6 vs. 1.4 ± 0.5, *p* = 0.04, n = 24). The open question revealed that none of the ST dogs and only two of the LT dogs (n = 24) were reported showing further unwanted behaviour. Details about the items and the shelter staff member’s judgement are displayed in Table 4. In particular, aggression towards dogs or unfamiliar people was considered to be a hindrance for rehoming a dog by shelter staff.

Regarding personality ratings, scores were possible on a scale from 1 “really does not describe this dog” to 6 “really does describe this dog”. LT dogs received statistically lower scores in the dimension “amicability” (mean ± SD: 3.5 ± 0.8 vs. 4.9 ± 0.8, *p* < 0.001, (Figure 1B) and tended to score lower on the dimension “training factor” (3.6 ± 0.8 vs. 4.3 ± 0.8, *p* = 0.013). LT dogs received higher scores in the dimensions “extraversion” (3.9 ± 1.2 vs. 2.9 ± 1.5, *p* < 0.001) and “motivation” (3.8 ± 0.9 vs. 3.1 ± 1.0, *p* < 0.001) than ST dogs. There was no difference between groups in the dimension “neuroticism” (2.6 ± 1 vs. 2.4 ± 0.9, *p* = 0.526).

### 3.2. Part II: Dog Assessment and Behavioural Observation

LT dogs did not differ in their approach behaviour (LT dogs no contact possible vs. ST dogs no contact possible: 10 vs. 5, *p* = 0.180) or signs of fear (LT dogs signs of fear vs. ST dogs signs of fear: 2 vs.1, *p* = 1) from ST dogs currently housed in the shelter. LT dogs tended to show more signs of offensive or defensive aggression than ST dogs currently housed in the shelter (10 vs. 5, *p* = 0.031).

The assessed parameters from the SQP were animal- and resource-based (more detailed information about the scoring can be found in the SQP by Barnard et al. (2014) [25]): kennel size, sharp edges, bedding type, number of beddings, dry/clean bedding, safe bedding, drinker type, drinker working, drinker safe, shelter of sun/rain/wind, air circulation, panting, shivering/huddling, diarrhoea, faeces visible, signs of pain, body condition, cleanliness of the dog, skin condition, and lameness. In all shelters, kennels were adequate, and no health or physical welfare problems were present in ST as well as LT dogs. Therefore, no statistical comparisons were carried out.

Overall, prior to feeding, the most common behaviours were resting head up or down, standing, walking and panting (Table 5). During resting, the most commonly observed behaviors were resting with head down or up and standing. The exploratory analysis of differences between groups showed that LT and ST dogs did not differ in their behaviour during the time before feeding, but during the resting period, dogs of the LT group spent more time resting with their head up, had more bouts of resting with the head down, and tended to spend more time circling and drinking than ST dogs (Table 5). The comparison between recording periods within group showed that during the time before feeding, LT dogs spent more time standing and vocalising, and they yawned more often. Conversely, time resting with head down was reduced. In addition, ST dogs spent less time resting with their head down, and they tended to drink and vocalise more in the pre-feeding period. For more detailed statistics and behaviours, see Supplementary Table S1.

## 4. Discussion

### 4.1. Part I: General and Morphological Characteristics, Problem Behaviour, and Personality

Overall, our results depict the following dog type as an LT dog: a neutered male dog of older age and larger size, being more likely to belong to the so-called “dangerous breeds”, and being less sociable, easily aroused, full of energy, and rather determined, displaying a range of problematic behaviours such as aggression towards other dogs or humans. This type of dogs seems to be at a high risk of staying in the shelter for 1 year or longer in an urban or semiurban environment when no-kill policies and breed-specific legislations are instituted.

Our result that dogs with a higher length of stay (LT dogs) were older was already shown before in the US [5], although there is evidence that this age dependency might only apply for mixed breed dogs [6]. On the other hand, Luescher and Medlock (2009) [37] did not identify age as a risk factor of becoming a long-term shelter dog. However, the demographics between the studies differ. In the study by Luescher and Medlock (2009) [37] in the US, no dog was older than seven years, whereas the mean age of dogs in the LT group in this study was above this age. Age might not be the pivotal characteristic that determines the length of stay in a shelter but automatically increases with the stay. Eventually, old age further reduces the adoption chances of LT dogs. Younger dogs might be less likely to have diseases and will therefore cause less expenditure for veterinary care than older dogs [5]. In addition, the decreased remaining lifespan of an older dog might be a reason for their overrepresentation in the LT group.

The result of our study, that more LT dogs are of larger size, is in accordance with previous US studies [5,6,7]. A higher popularity of small dogs might be related to a lower need for exercise [6], lower maintenance costs [11], and size restrictions set by a landlord [38]. Although smaller dogs were reported to show more unfavourable behaviour such as disobedience, excitement [39,40,41], and aggression [39], people may perceive these behavioural problems as less severe, more manageable, and tolerable in smaller dogs [6]. However, in our study, the size correlated with the breed. As there were more dogs of the “dangerous breeds” in the LT group and the size of these breeds started off at the size category L, this could be an additional factor contributing to the result of large dogs being at a higher risk of becoming a long-term shelter dog.

Similar to a finding in a US shelter, in our study, purebred dogs seemed to be preferred over mixed breed dogs [6]. People may connect certain behaviours with certain breeds [41] and think that the relative predictability of a purebred dog’s behaviour is higher [42], but appearance itself may play a role as well. However, also among purebred dogs, there is a difference in adoption chances between breeds [3,5,6,7]. The number of dogs of the “dangerous breeds” was significantly higher in the LT group than it was in the ST group. Lepper et al. (2002) [12] came to a similar result, that so-called “fighting breeds” have a prolonged length of stay in US shelters. As a result of breed-specific legislation in Vienna and Lower Austria, adopting a dog of a so-called “dangerous breed” involves restrictions of ownership. People who have been charged for any form of violence or drug abuse or for violation of the animal protection law are not allowed to have a dog of the listed breeds. The owner and the dog need to pass a basic obedience test to acquire a licence. If owner and dog repeatedly do not pass the test, the dog can be taken away from the owner. Furthermore, there is an obligation to permanently wear a muzzle and a leash in public places, and in Vienna, the owners are not allowed to consume alcohol when being in public with the dog [30,31]. These restrictions affect several aspects of owner and dog quality of life. It is understandable that this might discourage people to adopt a dog of the listed breeds, even if the dog does not show problematic behaviour, thereby increasing the risk of listed breeds of becoming a LT dog. Furthermore, dogs of so-called “dangerous breeds” may have a negative reputation linked to presumed higher levels of aggression [43]. Indeed, pit-bull-type dogs in American shelters received lower behavioural desirability and adoptability ratings from potential adopters than dogs of other breeds such as Labrador retriever and Border collie [43]. Already, the labelling of a dog as a member of a “dangerous breed” can prolong its stay in the shelter [43]. American studies have shown that only in 50% of the cases were dogs labelled as pit bulls actually members of that breed according to DNA breed signatures [44]. Generally, the identification of dog breeds, especially mixed breeds, based on visual identification, has a poor agreement with genetically determined breed heritage [45]. On the other hand, Brown et al. (2013) [5] could not confirm the prolonged stay of so-called “dangerous dogs” in their study in the US. However, the category “dangerous breed” in Brown’s study only comprised the four breeds American Bulldog, American Pit Bull Terrier, American Staffordshire Terrier, and Staffordshire Bull Terrier, whereas in this study, more breeds were included in the “dangerous breed” group. The different grouping and the difference in urbanity and breed preferences, i.e., the location of two of the shelters in middle-sized cities, might explain the differing results of the studies [46].

In contrast to other studies, our results did not confirm coat colour as a determinant for the length of stay in a shelter. Previous studies identified black-coloured, tan-coloured, and brindled dogs as having the worst adoption chances [12,14], whereas black and white, tricolour, merle, grey, and red individuals have the best chances for adoption in US shelters. However, Lepper et al. (2002) [12] and DeLeeuw (2008) [7] also identified the influence of the coat colour overall as of minor importance for adoption chances. In line with another study [7], an influence of coat length on the adoption chances was not confirmed in our study. However, in our dataset, information on coat length was missing in many cases. Therefore, this result should be regarded with caution.

In our study, LT dogs were more often males. Numerous studies from the US, UK, and Australia found females being preferred over males [8,9,11,12]. The reason could be that female dogs are thought to be easier to train, more obedient, and more demanding for affection than males [40]. Conversely, male dogs are thought to be more independent, going more astray, having a higher aggression potential and generally showing more behavioural problems [14]. However, behaviour is determined by many factors such as the breed [7,47] and owner–dog interactions [15,40,48]. The results of our study showed a difference in neuter status between groups with the long-term group having more neutered individuals than the short-term group, which seems to contradict previous studies from the US and Australia, indicating that neutered individuals are preferred over intact ones [8,9]. However, in our study, it was not possible to assess if the dogs were already neutered at the time of shelter intake. Therefore, it is difficult to draw conclusions if neuter status is a potential risk factor for becoming a long-term shelter dog or if the high proportion of neutered dogs in the LT group reflects management procedures. Most Austrian shelters do not routinely spay or neuter every dog admitted to the shelter [17]. It is more likely that long-term dogs have increased chances of being desexed during their stay in the shelter. However, the information about the neuter status was most often missing for dogs with a short length of stay in the shelter, and this might have led to an underestimation of neutered dogs in the ST group.

In contrast to previous studies conducted in the US and UK, the mode of relinquishment did not differ between the LT and ST group in this study [13,14]. In the US as well as in the UK, owner-surrendered dogs have better adoption chances compared to stray dogs [13,14]. Stray dogs might be less socialised as they had less close contact to humans [6]. According to the municipal authorities of Vienna responsible for veterinary services and animal welfare, no stray dog populations exist in Vienna (personal communication). Found dogs are most often abandoned or ran away and are used to close human contact.

Overall, LT dogs showed more problematic behaviours with higher scores for “aggression” and “high arousal” such as jumping and various forms of excitement than ST dogs. Titulaer et al. (2013) [24] also found that dogs with a length of stay in UK shelters with at least 6 months showed more signs of aggression, especially towards other dogs compared to short-term shelter dogs. This goes along with other studies stating aggression as the most frequent behavioural problem in US shelter dogs [10,49]. In addition to this, high arousal behaviours are among the most frequently reported undesirable behaviours by UK dog owners [15]. Furthermore, aggression and highly aroused behaviour are considered as major reasons for shelter relinquishment of dogs [10,50]. Our results indicate that it is highly likely that LT dogs show a multitude of undesired behaviours reducing their adoption chances.

Regarding personality assessment, dogs of the LT group were ranked lower in the dimension “amicability” and tended to be ranked lower in the dimension “training factor”. LT dogs were ranked higher in the dimensions “extraversion” and “motivation” than dogs of the ST group. Studies confirmed personality as one of the most important factors influencing an adopter’s choice in the US [2]. Australians describe their ideal companion dog as friendly, sociable, obedient, affectionate, calm/compliant, energetic, and non-aggressive [16]. These named personality traits coincide with the adjectives building up the dimension “amicability” (e.g., easy-going, friendly, non-aggressive, relaxed, sociable) and “training factor” (e.g., obedient, attentive). The lower ranking of the LT dogs in these personality dimensions fits the view of the raters, i.e., shelter staff, that these dogs are seemingly more aggressive and less obedient as reported in the assessment of behavioural problems. LT dogs were also scored higher in the dimension “motivation”, which included attributes such as assertive, independent, determined, and tenacious, as well as the dimension “extraversion”, which included traits such as active, energetic, and lively but also excitable, hyperactive, and restless. These personality traits can become problematic in combination with a low ranking in the dimension “training factor”, possibly resulting in highly aroused and disobedient behaviour.

Overall, the results of the personality assessment point into the same direction as the results of the behavioural problem assessment, indicating that aggression and high arousal behaviour seem to be of great importance for adoptability and the decision making of adopters so that LT dogs rated less favourable in these attributes have lower chances to be rehomed. However, this study does not allow any conclusion about the causality of problematic behaviours, i.e., if dogs became long-term shelter residents due to these unwanted behaviours or if the long-term confinement in a shelter influenced the dog’s behaviour. Depending on the circumstances in a shelter, a dog’s (problem) behaviour could get worse or improve. A restricted spatial and social environment can elicit or enhance stereotypies [18], whereas environmental alterations and behavioural modification training can improve a dog’s behaviour and thereby its adoption chances [37]. Behavioural problems and personality questionnaires were completed by the same person (the person that was most familiar with the dogs in question). These questionnaires reflect the subjective perception of that person regarding the dogs’ behaviour and personality. For comparison, we only chose rehomed ST dogs the shelter staff persons could remember. The memories about these dogs might not have been as accurate as for the currently shelter housed LT dogs. Furthermore, the memory of the staff might have been biased towards friendly, easy-going, or attractive dogs. Although we carefully tried to avoid creating bias, we cannot fully exclude the possibility that a bias towards a more positive rating of ST dogs compared to LT dogs might be present in our data.

Overall, becoming a long-term shelter dog seems to be mostly determined by behavioural and breed characteristics. Given that a shelter has the resources to institute a behaviour modification program, the behaviour of dogs can be improved so that adoptability increases. However, breed cannot be changed. The contribution of breed-specific legislation to those dogs becoming long-term shelter residents needs to be determined.

### 4.2. Part II: Dog Assessment and Behavioural Observation

A dog showing affiliative behaviour towards potential adopters has been reported to lead to better adoption chances in the US compared to dogs that are not actively seeking contact to the human [2]. In contrast to the findings by Arhant and Troxler (2014) [27], the approach behaviour of dogs did not decrease with an increase of length of stay in a shelter in Austria. It is possible that this study could not confirm this finding due to the small sample size. Initially, it was planned to conduct the study with 35 matched dog pairs. Due to practical limitations, we were not able to recruit this number of dog pairs. The reduced sample size and reduced statistical power limited detecting smaller effects and applies to the entire part II that has been conducted with 20 dog pairs only.

Although LT and ST dogs were similar regarding their interest in a human approaching their kennel (number of dogs with “contact possible” not statistically different), more LT than ST dogs showed signs of defensive or offensive aggression towards the person kneeling and talking to the dog. Looking more closely at the test procedure with its two parts and the categorisation of dog behaviour used to assess reaction towards unfamiliar humans, the results of the two scores might lead to different conclusions. The first part of the unfamiliar person test [27] looks at the willingness of a dog to explore a human near their enclosure, including dogs showing subtle signs of fear or aggression in the category “contact possible” if the dog approaches and shows interest in the person. Conversely, “no contact”, including driving the human away, is shown by intense fear-related or aggressive behaviours, or apathy/depression-like states. The second part of the test procedure differentiates between signs of fear and aggression including subtle as well as more intense signs [25]. These behaviours are reflecting the animal–human relationship [25] and are mainly determined by experiences an individual has with humans [26]. Signs of fear or aggression indicate a negative emotional state towards the presence of (unfamiliar) humans. However, dogs totally avoiding interactions with humans (categorised as “no contact” during the first part) could, in addition, suffer from chronic stress caused by an unpredictable or overstimulating environment, exacerbating the fear of humans. This could lead to depression-like states that are proposed to be associated with decreased welfare [51]. In our sample, half of the LT dogs were categorised as “no contact”, but this was not significantly different from ST dogs. We conclude that the reaction towards humans did not show obvious effects of increased chronic stress in LT dogs in this sample, but a negative animal–human relationship was present in LT dogs more often.

The Shelter Quality Protocol assesses animal-, resource-, and management-based parameters in order to evaluate the welfare of dogs in shelters. It was developed as a tool to assess the welfare of sheltered dogs related to the management of stray dog populations in urban areas. In this study, we only assessed resource- and animal-based parameters that mainly reflect the physical wellbeing in individual shelter dogs [25]. The physical welfare status according to the assessed parameters was high in all shelters, and there were no differences between short-term and long-term housed dogs. However, it was reasonable to expect differences in the psychological welfare rather than the physical welfare, as all dogs were provided with food, water, medical attention, and exercise. The finding that the length of stay in the shelter does not necessarily impair a dog’s physical welfare is in accordance with findings by Titulaer et al. (2013) [24] and Wells et al. (2002) [4]. Considering that the protocol was developed for the management and long-term sheltering of stray dog populations in urban areas, it is possible that the dog populations and standards of Austrian shelters do not comply with the SQP and that this tool is not appropriate to detect differences in the dogs’ physical welfare in this context. Furthermore, it is difficult to draw the line between a long-term shelter dog and a short-term shelter dog. Our classification of the ST group with a length of stay up to 5 months might have been too broad. It is possible that dogs having spent up to 5 months in a shelter can already be considered as long-term shelter dogs, and their welfare could therefore be comparable to our LT dog population.

Behaviour is a sensitive indicator for dog welfare, especially in the shelter environment [52]. During the resting period, LT dogs were resting longer with their head up and had more bouts of resting with their head down, although the duration of resting with head down was similar to ST dogs. In dogs, a resting position with the head down indicates sustained rest [53], whereas a resting position with the head up indicates a state of alertness [54]. The higher number of bouts of resting with the head down might indicate that the periods of sustained rest in LT dogs were more often disturbed compared to ST dogs. In addition, LT dogs tended to circle and drink more during resting periods. This might lead to the conclusion that LT dogs are less able to relax during the resting periods than ST dogs. This is supported by the results of a recent study. Shelter dogs that rested less during the day and showed more repetitive behaviour had a more pessimistic judgment bias [23]. These differences in behaviour between individuals in the same environment could reflect differences in susceptibility to become aroused and may reflect the psychological welfare status of individuals [23]. However, LT dogs in our study were easily aroused also in other contexts. Therefore, we cannot disentangle the effect of personality from the effect of environment.

The comparison of the periods showed that LT as well as ST dogs spent less time resting with head up in the pre-feeding period. Furthermore, LT dogs spent more time standing, vocalising, and they yawned more often before feeding than in the resting period. The behaviour of ST dogs was less influenced by the period. However, there was a statistical tendency to vocalise and drink more often before being fed. These behaviours very likely indicated high arousal and were found to be associated with an acute stress response when being challenged [55]. Furthermore, they can be interpreted as anticipatory behaviour for being fed. In general, anticipation of food is considered to be positively valanced, but anticipation can easily result in frustration if control over the food resource is not provided [56]. The reason why ST dogs reacted less might be that ST dogs are not as familiar with the shelter routine and therefore show less anticipatory behaviour. However, Wells and Hepper (1992) [14] found behavioural changes in shelter dogs after five to six days after admission, indicating that dogs should have become familiar with their new surrounding and routine. As dogs of the ST group had a minimum length of stay of two weeks, they should have become familiar to the daily feeding routine by then. An alternative interpretation is that LT dogs are more at risk to display high arousal behaviour in challenging situations than short-term sheltered dogs, which may be due to an increased reactivity to stimuli that might act as acute stressors. This again is in accordance with our finding of a higher susceptibility of LT dogs to show problem behaviour related to high arousal.

Overall, it is difficult to tell apart whether the shelter stay induced differences in behaviour or whether dog personality made LT dogs more susceptible to stressors and increased their risk to show high arousal behaviours either in the shelter or already before entering the shelter. Personality also affects susceptibility to suffer from acute or chronic stress [57]. It is highly likely that being prone to be easily aroused in challenging situations and showing a tendency to cope with an aggressive response will increase the risk of relinquishment as well as the risk of a long-term shelter stay in a shelter. However, we adopted an exploratory approach for the analyses of behavioural observations, and the results were not corrected for multiple testing and should be regarded with caution.

## 5. Conclusions and Implications

Our study identified distinct characteristics that were more common in long-term shelter dogs. Behaviour problems that were more prominent in long-term shelter dogs were related to aggression and high arousal. It is important to prevent the worsening of these problem behaviours, as this may reduce adoption chances even more.

Our study did not find a negative effect of a stay above one year on the welfare of dogs regarding physical welfare as assessed with the Shelter Quality Protocol. However, the results of behavioural observations gave rise to the assumption that long-term shelter dogs might be more affected by stressors, e.g., leading to highly aroused behaviour and difficulties to relax in the shelter environment. Although it is difficult to disentangle the effects of personality and environment, we suggest providing especially long-term shelter dogs with a predictable environment, a structured enrichment program, and undisturbed resting periods in order to manage arousal levels.

The results obtained in this study can be used in the short run for the early identification and enhancement of the promotion of dogs being at risk of becoming a long-term shelter dog. Based on the individual dog’s behaviour and the risk its behaviour problems pose to the public, different strategies to manage those dogs are necessary. Dogs that present with obedience problems due to high arousal very likely will profit from behaviour modification programs. For dogs that present with aggression problems, the prognosis of behaviour modification and the risk to the public need to be carefully evaluated before considering rehoming these dogs. Other long-term management options need to be discussed and evaluated in case of rehoming being too risky. Future studies are urgently needed to develop effective strategies how to deal with and ideally prevent the development of long-term shelter populations in no-kill shelters. Furthermore, the effects of breed-specific legislation on adoption preference should be evaluated.

## Figures and Tables

**Table 1:** Ethogram of the observed behaviours. For behaviour type “state event”, duration and frequency are recorded, whereas for behaviour type “point event”, only the frequency is recorded.

Behaviour	Behaviour Type	Description	Reference Source
Circling	State event	Dog walks around in small circle	[22]
Self-licking	State event	Dog licks or chews its own body	Adapted from [18]
Drinking	State event	Dog takes up water	
Panting	State event	Dog pants for reasons other than physical exertion observable breathing with an open mouth	[13], adapted from [21]
Vocalisation	State event	Dog barks. New bout starts after 3 s break	Adapted from [18]
Hiding	State event	Dog is obscured from the view by actively seeking a place that provides visual cover from 4 sides	[22]
Chewing bars or kennel inventory	State event	Dog chews and bites at the wire of the kennel or anything in it not for the purpose of biting. New bout after 3 s break	Adapted from [18]
Licking kennel	State event	Dog licks kennel walls, floor, or any items without removing any liquids or food. New bout after 3 s break	Adapted from [13]
Stretching	State event	Extending body and one or more front and/or hind-legs while remaining stationary	[13]
Paw lifting	Point event	At least one paw is lifted up from the floor for at least 3 s	[21]
Yawning	Point event	Dog opens the mouth and inhales (yawning 1 min before and after resting is excluded)	Adapted from [13]
Standing on hind legs	State event	Dog jumps or climbs up kennel wall or door, standing on the hindlegs	
Sitting	State event	Dog is sitting half or fully on its hip	
Resting head up	State event	Dog is laying on the floor. Head is held above the ground	
Resting head down	State event	Dog is laying on the floor. Head is resting on the ground. Kennel inventory or its paws	
Sniffing	State event	Dog inhales air through the nose with a closed mouth close (5 cm) to a surface	
Mouth licking	Point event	Dog licks mouth with at least 1/3 of the tongue visible (1 min after drinking is excluded). Smacking is not counted	
Walking	State event	Dog moves all 4 legs and changes its position in the room. New bout starts after 2 s where no leg was moved	
Standing	State event	Dog stays stationary. Dog may change its position but only by moving up to three legs	
Not visible	State event	Dog is obscured from observer by being hidden behind furniture when strolling around the kennel	

**Table 2:** Crosstab of the general and morphological characteristics of short-term shelter dogs (ST) and long-term shelter dogs (LT) groups. The ST group only included already rehomed dogs with a maximum length of stay of five months. Due to correction for multiple testing with Bonferroni correction for 15 tests, Chi^2^ tests are considered significant with *p* ≤ 0.003 and as a trend with 0.003 < *p* ≤ 0.006. Significant results are shown in bold and trends in italics. Standardised residuals (SR) greater or smaller than 2 or −2 are marked with bold numbers. For these characteristics, the observed frequency deviates from the expected frequency.

	LT			ST			Total
	Frequency	Expected Frequency	SR	Frequency	Expected Frequency	SR	Frequency
**Breed group:** **Chi^2^ = 131.06, *p* < 0.001**							
purebred	29	30.7	−0.3	250	248.3	0.1	279
Mix	44	83.2	−4.3	711	671.8	1.5	755
Dangerous breeds	58	17.1	9.9	97	137.9	**−3.5**	155
Total	131			1058			1189
**Sex:** **Chi^2^ = 16.08, p < 0.001**							
male	92	71.1	2.5	440	460.9	−1.0	532
female	35	55.9	−2.8	383	362.1	1.1	418
Total	127			823			950
**Neuter Status:** **Chi^2^ = 142.52, *p* < 0.001**							
spayed/neutered	69	24.1	9.1	56	100.9	−4.5	96
not spayed/neutered	19	63.9	−5.6	312	267.1	2.7	204
Total	88			368			456
**Mode of relinquishment:** *Chi^2^ = 3.229*, *p* = *0.358*							
find	36	33.9	0.4	264	266.1	−0.1	300
owner relinquished	81	80.0	0.1	628	629.0	0.0	709
confiscated	18	18.4	−0.1	145	144.6	0.0	163
birth	0	2.7	−1.6	24	21.3	0.6	24
Total	135			1061			1196
**Coat colour:** *Chi^2^ = 4.015, p* = *0.260*							
light	36	31.0	0.9	244	249.0	−0.3	280
dark	53	55.1	−0.3	445	442.9	0.1	498
multicolour	42	47.3	−0.8	386	380.7	0.3	428
brindled	5	2.7	1.4	19	21.3	−0.5	24
Total	136			1094			1230
**Hair length:** *Chi^2^ = 12.016*, *p* = *0.017*							
short hair	76	65.4	1.3	269	279.6	−0.6	345
long hair	7	18.2	−2.6	89	77.8	1.3	96
wirehaired	0	0.9	−1.0	5	4.1	0.5	5
curly	1	0.8	0.3	3	3.2	−0.1	4
medium	17	15.7	0.3	66	67.3	−0.2	83
Total	101			432			533
**Size:** **Chi^2^ = 80.290, *p* < 0.001**							
XXS	1	20.7	−4.3	118	98.3	2.0	119
XS	6	21.7	−3.4	119	103.3	1.5	125
S	8	12.2	−1.2	62	57.8	0.5	70
M	13	4.7	3.8	14	22.3	−1.8	27
L	55	33.6	3.7	138	159.4	−1.7	193
XL	27	25.4	0.3	119	120.6	−0.1	146
XXL	19	10.8	2.5	43	51.2	−1.1	62
Total	129			613			742

**Table 3:** Descriptive statistic of age and age when being admitted for LT and ST dogs including dogs for which no information about the age was available. The ST group only included already rehomed dogs with a maximum length of stay of five months.

	ST				LT			
Age [y]	N	%	N Admitted	% Admitted	N	%	N Admitted	% Admitted
<1	171	15.4	184	16.6	0	0	13	9.2
1–3	112	10.1	121	10.9	12	8.5	37	26.2
3–6	81	7.3	76	6.8	27	19.1	33	23.4
6–9	53	4.8	42	3.8	29	20.6	12	8.5
>9	40	3.6	34	3.1	33	23.4	6	4.3
unknown	654	58.9	654	58.9	40	28.4	40	28.4

**Table 4:** Analysis of the single problem behaviour items. Scores on a four-point scale were 1 = never, 2 = occasionally, 3 = sometimes, 4 = frequently. Analyses of the single behaviour items were not corrected for multiple testing to identify outstanding behaviours that differ between LT and ST dogs. Significant results are in bold and trends are in italics. The “Problem for adoption” column shows the percentage of behaviours caretakers judged as a problem for adoption. N = 24.

Behaviour	Group	Mean	SD	Min	Max		*p*	Problem for Adoption in %	*p*
Aggression towards dog	ST	1.3	0.6	1	3			4	**0.001**
	LT	2.9	1.0	1	4		**<0.001**	56	
Aggression towards familiar	ST	1.0	0.2	1	2			0	*0.063*
	LT	1.6	0.8	1	4		**0.004**	20	
Aggression towards unfamiliar	ST	1.1	0.3	1	2			0	**0.001**
	LT	2.3	1.3	1	4		**0.001**	44	
Redirected aggression	ST	1.0	0.3	0	2			4	1.000
	LT	1.8	1.1	1	4		**0.005**	28	
Guarding food bowl	ST	1.2	0.5	1	3			0	0.500
	LT	1.9	1.0	1	4		**0.006**	12	
Jumping	ST	2.0	1.0	1	4			8	0.125
	LT	2.9	1.3	1	4		**0.031**	24	
Excitement food	ST	1.8	1.0	1	4			0	0.500
	LT	2.4	1.0	1	4		**0.001**	8	
Excitement unfamiliar	ST	1.6	0.8	1	3			0	**0.008**
	LT	2.9	1.1	1	4		**0.001**	32	
Excitement familiar	ST	1.6	0.8	1	3			0	**0.031**
	LT	2.4	1.0	1	4		**0.001**	24	
Excitement walk	ST	1.9	0.9	1	4			0	**0.016**
	LT	2.5	0.8	1	4		**0.025**	28	
Pulling on the leash	ST	2.0	1.0	1		4		4	*0.070*
	LT	2.8	1.0	1		4	**0.005**	8	
Chasing (e.g., cars, joggers)	ST	1.2	0.6	1		3		8	0.625
	LT	1.4	0.8	1		4	0.238	8	
Mouthing hands or clothes	ST	1.3	0.6	1		3		4	1.000
	LT	1.6	0.9	1		4	0.142	12	
Sexual behaviour towards people	ST	1.2	0.8	1		5		0	0.500
	LT	1.3	0.5	1		3	0.332	4	
Hiding familiar people	ST	1.2	0.4	1		2		4	1.000
	LT	1.3	0.7	1		4	0.414	4	
Hiding unfamiliar	ST	1.4	0.8	1		4		0	*0.063*
	LT	1.6	1.1	1		4	0.351	20	
Fear from noise	ST	1.9	0.9	1		4		0	1.000
	LT	2.2	0.9	1		4	0.106	8	
Fear from dogs	ST	1.2	0.4	1		2		0	0.500
	LT	1.4	0.5	1		2	0.157	4	
Destructive behaviour	ST	*1.5*	*0.7*	*1*		*4*		*4*	0.375
	LT	*1.9*	*1.1*	*1*		*4*	*0.053*	*16*	
Barking	ST	1.8	1.0	1		4		4	1.000
	LT	2.2	0.9	1		4	0.123	4	
Repetitive behaviour	ST	1.0	0.2	1		2		0	1.000
	LT	1.4	0.9	1		4		8	

**Table 5:** Analysis of the video recordings comparison between groups. TD = total duration, NO = number of occurrences per 10 min, corrected for the time visible. Significant results are in bold and trends are in italics.

		ST Feeding vs. Resting	LT Feeding vs. Resting	ST vs. LT	ST			LT		
		*p*	*p*	*p*	Median	Min	Max	Median	Min	Max
TD walking	Feeding	0.12	0.18	0.28	40.1	0.0	247.3	29.9	0.0	149.5
	Resting			0.23	17.6	0.0	188.0	15.1	1.9	95.7
TD standing	Feeding	0.57	**0.01**	0.79	143.8	0.0	532.9	122.2	0.0	599.2
	Resting			0.17	85.5	0.0	550.8	59.5	0.0	339.8
TD sniffing	Feeding	0.81	0.98	0.10	3.0	0.0	40.3	1.9	0.0	42.2
	Resting			0.75	0.0	0.0	42.9	1.6	0.0	84.8
TD drinking	Feeding	*0.08*	0.50	0.67	0.0	0.0	4.8	0.0	0.0	7.0
	Resting			*0.07*	*0.0*	*0.0*	*6.2*	*0.0*	*0.0*	*21.4*
TD panting	Feeding	0.20	*0.10*	0.59	33.8	0.0	521.9	42.9	0.0	487.4
	Resting			0.73	0.0	0.0	470.3	1.3	0.0	434.4
TD vocalisation	Feeding	*0.08*	**0.02**	0.76	7.8	0.0	461.8	9.8	0.0	164.3
	Resting			0.28	4.0	0.0	199.0	0.6	0.0	39.5
TD chewing	Feeding	0.27	0.18	1.00	0.0	0.0	30.3	0.0	0.0	523.9
	Resting			0.11	0.0	0.0	76.2	0.0	0.0	0.0
TD licking	Feeding	0.72	0.67	0.72	0.0	0.0	14.5	0.0	0.0	55.0
	Resting			0.11	0.0	0.0	0.0	0.0	0.0	28.6
TD circling	Feeding	0.32	0.14	0.11	0.0	0.0	0.0	0.0	0.0	67.6
	Resting			*0.08*	*0.0*	*0.0*	*2.5*	*0.0*	*0.0*	*10.7*
NO yawning	Feeding	0.37	**0.03**	0.24	0.5	0.0	1.5	0.5	0.0	4.0
	Resting			0.32	0.0	0.0	4.5	0.0	0.0	1.0
TD sitting	Feeding	0.47	0.36	0.80	1.2	0.0	412.7	12.3	0.0	439.7
	Resting			0.26	14.2	0.0	426.7	38.4	0.0	183.9
TD resting head up	Feeding	0.75	0.31	0.31	61.0	0.0	283.8	134.8	0.0	427.1
	Resting			**0.03**	36.1	0.0	278.5	160.2	0.0	429.5
TD resting head down	Feeding	**0.01**	**0.01**	0.15	151.0	0.0	586.6	53.4	0.0	370.9
	Resting			0.97	252.8	0.0	600.2	240.0	0.0	532.6
NO resting head down	Feeding	0.35	0.49	0.80	2.0	0.0	7.0	1.5	0.0	9.4
	Resting			**0.02**	1.0	0.0	3.5	2.5	0.0	8.0

## Data Availability

The data presented in this study are available in Supplementary Materials Excel S1: Study Data.

## Supplementary Materials

The following are available online at https://www.mdpi.com/2076-2615/11/1/194/s1, Excel S1: Study Data, Table S1: Analysis of the video recordings comparison between groups.
